# Figure-Eight Coils for Magnetic Stimulation: From Focal Stimulation to Deep Stimulation

**DOI:** 10.3389/fnhum.2021.805971

**Published:** 2021-12-16

**Authors:** Shoogo Ueno, Masaki Sekino

**Affiliations:** ^1^Department of Biomedical Engineering, Graduate School of Medicine, The University of Tokyo, Tokyo, Japan; ^2^Department of Bioengineering, Graduate School of Engineering, The University of Tokyo, Tokyo, Japan

**Keywords:** transcranial magnetic stimulation, figure-eight coil, brain stimulation, brain mapping, depth-focality tradeoff

## Abstract

This article reviews the evolution and recent developments of transcranial magnetic brain stimulation using figure-eight coils to stimulate localized areas in the human brain. Geometric variations of figure-eight coils and their characteristics are reviewed and discussed for applications in neuroscience and medicine. Recent topics of figure-eight coils, such as focality of figure-eight coils, tradeoff between depth and focality, and approaches for extending depth, are discussed.

## Introduction

Transcranial magnetic stimulation (TMS) is a technique employed for the transcranial stimulation of the human brain using a coil positioned on the surface of the head. The coil is driven by pulsed electric currents of several hundred amperes for approximately 50–150 μs to produce transient magnetic fields of approximately 1 T. This induces electric fields in the brain in accordance with Faraday’s law. The induced electric fields influence neurons in the brain when the neurons receive some level of electric excitation. TMS was first reported over three decades ago in a study using a circular coil (Barker et al., 1985), in which a recordable electromyography response was elicited from the stimulation to the motor cortex (MC). This successful demonstration greatly impacted the scientific community. However, it was difficult to locally stimulate the targeted areas in the brain using a circular coil. Subsequently, a method of localized brain stimulation with a figure-eight coil was proposed (Ueno et al., 1988), and the stimulation of the human MC within a 5-mm resolution was achieved (Ueno et al., 1990).

Implementing TMS using a figure-eight coil is advantageous in the localized stimulation of the brain. This has led to a rapid expansion in the study of functional brain research; studies concerning the functional organization of the human brain, neuronal dynamic connectivity, and neuronal plasticity in the cortex have been successfully conducted using figure-eight coils. In view of its excellent performance in localized brain stimulation, TMS with a figure-eight coil is now widely used in basic and clinical medicine (Ueno, 2021).

Building on the success of TMS, repetitive TMS (rTMS) with repetitive pulsed stimulation was introduced in clinical medicine as a potential treatment for pain, depression, Parkinson’s disease, and neurorehabilitation. To treat these ailments, specific areas in the deeper parts of the brain need to be stimulated. To achieve this type of deep brain stimulation, several coil configurations were developed (Roth et al., 2002; Zangen et al., 2005; Crowther et al., 2011; Lu and Ueno, 2015, 2017).

In this article, we introduce TMS using a figure-eight coil and structural variations of figure-eight coil, as well as other coil configurations for surface and deep brain stimulation. We discuss the advantages and limitations of these coil configurations, focusing on the focality, tradeoff between depth and focality, and approaches for extending depth.

## Focality of Figure-Eight Coils

**FIGURE 1:**
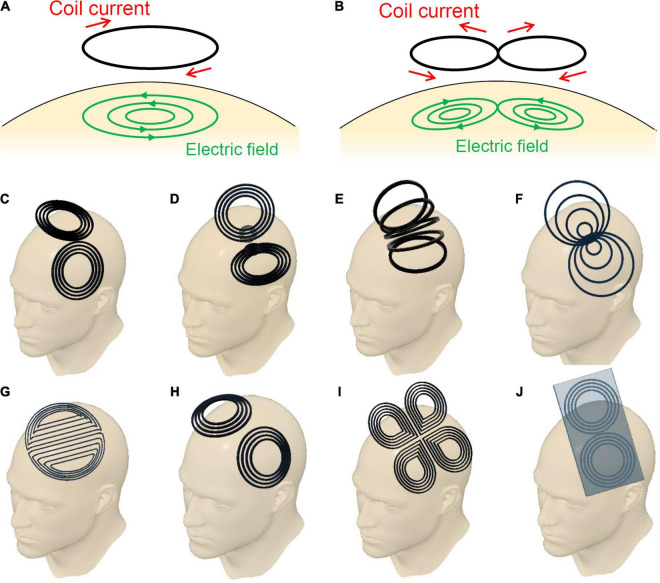
**(A)** Circular coil, **(B)** planar figure-eight coil, **(C)** butterfly coil, **(D)** quadruple butterfly coil, **(E)** slinky coil, **(F)** eccentric figure-eight coil, **(G)** double-D coil, **(H)** H6-coil, **(I)** cloverleaf coil, and **(J)** figure-eight coil with iron core.

When we use a round coil, induced electric fields in the brain flow in a concentric manner as shown in Figure 1A. The intensity of the electric fields increases in proportion to the radius; the intensity is zero at the center of the coil, and the maximum at the edge of the coil. In contrast, when we use a figure-eight coil, induced electric fields flow in the brain, making two vortices, as shown in Figure 1B. The two vortices merge at the center of the figure-eight. Thereby we can deliver localized and focal brain stimulation (Ueno et al., 1988, 1990). Our computer simulation showed that the electric fields at the target is three times higher than those at non-target areas (Ueno et al., 1988; Sekino and Ueno, 2004a,b).

Since the induced electric fields, or, induced electric currents, flow in the direction of the tangent of coils, the direction of stimulating currents is controlled by rotating the coil. The direction-controlled magnetic stimulation, or, so-called vectorial magnetic stimulation is carried out by changing the amplitude and direction of stimulating currents.

The localized and direction-controlled stimulation of neurons in the brain is useful in studying both anatomical and functional organizations of the brain. The pyramidal neurons in the cortex are more easily excited when the stimulating electric fields, or, stimulating electric currents, flow in parallel to the axons of the pyramidal neurons, compared with the stimulation by currents which flow in the direction perpendicular to the axons. The neuronal excitation is caused by transmembrane potentials across the cell membrane. The transmembrane potential is caused by the outward currents out of the neurons, which results in the depolarization of the membrane. When the depolarization exceeds a threshold of the excitable tissue, the neuron is excited. The outward currents are driven by the activating function, or, the negative value of spatial gradient of induced electric fields (Basser et al., 1992).

Therefore, TMS with a figure-eight coil has the advantages in targeted and vectorial stimulation of the brain, compared with those with round coil configurations.

## Structural Variation of Figure-Eight Coils

Since the invention of the figure-eight coil, researchers have constantly attempted to improve its performance. The focus of these attempts has been the geometry of the coil windings, since it greatly affects the performance of the coil. Over the years, various modifications of the coil geometry and their resulting advantages have been reported. Figure 1 summarizes the major variations in the design of the figure-eight coils. Figure 1B shows a simple planar coil consisting of a pair of circles, which is the most basic form among the variants. The induced electric field converges directly below the center of the coil, which exhibits a locally high electric field intensity. Butterfly coils or double cone coils, shown in Figure 1C, are now widely used in clinical applications. The coil is bent at an acute angle at the center between the left and right wings. The bending forces the coil to conform to the shape of the human head. Moreover, it results in an increased depth of the induced electric fields in the brain. The quadruple butterfly coils illustrated in Figure 1D are also bent at the center of the coil, but at an obtuse angle (Rastogi et al., 2017). This coil achieves a high electric-field intensity at the intersection of the wings. Additionally, the bent shape leads to a reduction in the current density in the surrounding areas. Thus, this coil enhances the focality of the induced electric field. Slinky coils, illustrated in Figure 1E, are another variation of figure-eight coils (Krasteva et al., 2002). Because of the contributions of coil elements having obtuse angles, the coil provides electric field distributions with an enhanced focality. Eccentric figure-eight coils, shown in Figure 1F, can achieve an enhanced electric field intensity owing to their modified in-plane winding geometry (Sekino et al., 2015). The winding centers of the left and right coils are shifted toward the middle, forming a dense coil conductor in the middle. Its high efficiency in inducing electric fields, leads to downsizing of the driving circuit. Moreover, the dense conductors in the middle enhances the focality of stimulation. Double-D coils, shown in Figure 1G, are intended for the stimulation of wider areas of the brain (Sekino et al., 2017). This coil has a deformation in the direction opposite to that of the eccentric figure-eight coils. The double-D coils have straight conductors in the middle, with gaps between the conductors. Because of the extended area of stimulation, the stimulating effect is more stable despite the small displacements of the coil that naturally occur during repeated stimulations. H-coils offer a series of coil geometries designed for generating electric fields being deeper than those of typical TMS coils which only stimulate more superficial layers of the cortex (Tendler et al., 2016). As shown in Figure 1H, several coil geometries in the H-coil series are based on figure-eight coils. These H-coils have characteristics similar to those of butterfly coils, and are now widely applied in the treatment of depression. Figure 1I presents a combination of two figure-eight coils that generate electric fields in orthogonal directions (Rotem et al., 2014). When biphasic pulse currents are applied to the two coils with a phase shift of a quarter cycle, the resulting electric field rotates in the coil plane. The excitability of cortical tissues depends on the direction of the electric field. Thus, the rotating electric fields provide stable stimulating effects regardless of the coil orientation. The electric power efficiency of figure-eight coils can be improved by using an iron core, as illustrated in Figure 1J. This enables the generation of strong magnetic fields with smaller driving currents (Yamamoto et al., 2016). The improvement of power efficiency is beneficial for therapeutic applications that require repeated stimulations with frequencies over 5 Hz. However, the iron core should be designed to minimize eddy currents in the core.

## Tradeoff Between Depth and Focality

As electromagnetic fields generated from a coil attenuate with increasing distance from the coil, superficial areas in the brain, such as the cerebral cortex, are more strongly stimulated by TMS. This leads to excitation or inhibition of deeper regions in the brain depending on the functional connectivity in the brain. Such neuromodulation of the deeper regions is considered to be the key to obtaining therapeutic effects. Extensive research and development has been conducted to increase the depth of electric fields. Several clinical studies on TMS treatment have conclusively demonstrated that deeper stimulations achieve better therapeutic effects (Shimizu et al., 2017). However, direct stimulation of deeper regions of the brain continues to be one of the biggest technical challenges in TMS.

**FIGURE 2:**
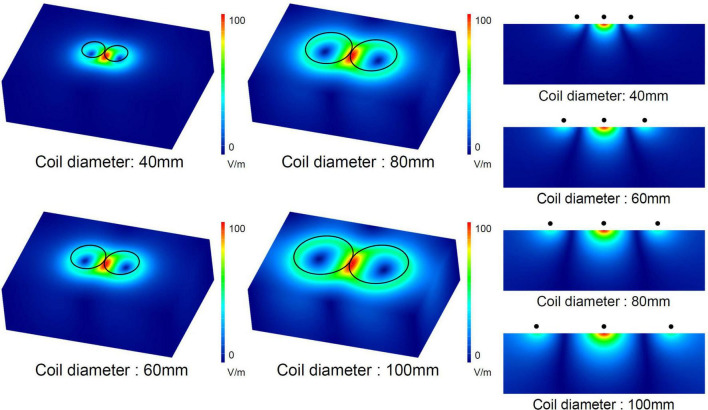
Numerical simulation of electric fields induced by figure-eight coils with four different diameters. The electric current in the coil was 1 kA for the four diameters.

Figure 2 shows a numerical simulation of electric currents induced by figure-eight coils with four different diameters ranging from 40 to 100 mm. The increase in the coil diameter leads to an increased depth of the induced electric fields, suggesting that larger coils are suitable for achieving deeper stimulations. However, larger coils exhibit an extended area of stimulation, which leads to stimulation outside the target area, increasing the potential risk of side effects. To provide effective and safe TMS, coils with both depth and focality are preferred. This simulation shows that there is a trade-off between depth and focality in induced electric fields.

A comparison of the electric fields in the human brain induced by a butterfly coil, H-coil, Halo coil, and planar figure-eight coil was conducted in a study (Lu and Ueno, 2017). The butterfly, H-, and Halo coils have significantly deep field penetrations compared to the planar figure-eight coil, at the expense of focality. The intensities of electric fields in the thalamus were 86.2, 28.7, 47.7, and 21.7 V/m for the butterfly, H-, Halo, and planar figure-eight coils, respectively. Interestingly, the butterfly and Halo coils are more adept at stimulating deep brain subregions compared to the H-coil. Therefore, the butterfly coil has an appropriate balance of depth and focality.

A systematic benchmarking of TMS coils was conducted by comparing the electric field distributions of 50 different coils (Deng et al., 2013). The results showed that there is a clear tradeoff between depth and focality, and coils with deeper penetrations exhibited wider stimulated areas. The figure-eight and H-coils are most suitable for focused and wide stimulations, respectively. The butterfly coil exhibited both moderate depth and focality. A significant observation of this study was that the bending angle between the two wings of the butterfly coil affected the balance between depth and focality. In the study, the maximum field depth was obtained at a bending angle of 110°.

## Recent Approaches to Extending Depth

Realizing the importance of deeper penetration of the induced electric field in TMS, recent studies have proposed novel approaches to this problem.

Mathematical methodologies for solving inverse problems play an important role in biomagnetics, specifically magnetoencephalography. These methodologies have recently been applied to the coil design of TMS. In the history of TMS, researchers have invented various coils. The characteristics of these coils can be compared to determine which of them is best suited to specific applications. Inverse analysis provides a framework for optimizing the geometry and size of coil windings for a given target electric field distribution in the brain (Peeren, 2003; Liu et al., 2020). A hypothetical potential is defined on a two-dimensional curved surface on which the coil windings are formed. Partial derivatives of the potential provide current distributions in the coil. Biot–Savart’s law and Faraday’s law of induction describe a linear relationship between the potential on the coil surface and the electric field distribution inside the plane. A cost function is defined as the spatially integrated difference between the given target electric field and the electric field generated from the coil potential. Then, the optimum coil potential for the target electric field can be obtained by minimizing the cost function. Notably, a focused target electric field led to a solution of figure-eight coils, although the algorithm did not have prior knowledge of figure-eight coils (Liu et al., 2020). This result provides evidence from a mathematical viewpoint that a figure-eight coil is feasible for providing focal stimulation. This framework will be used in the future to systematically study coil designs to realize deeper brain stimulations.

A recent study showed the concept of temporally interfering electric stimulation, which uses a pair of electric fields with slightly different frequencies (Grossman et al., 2017). Simultaneous application of the two electric fields generates an envelope of waveforms that vary with the frequency difference between the two carrier frequencies. When the carrier frequencies exceeded 10^3^ Hz, the neurons were unresponsive. However, the envelope frequency is approximately 10 Hz, which induces neuron excitation. Interestingly, the maximum magnitude of the envelope may appear in the deep regions of the brain. This technique enables the local stimulation of deep regions from electrodes attached to the surface of the head. The concept of temporal interference can also be introduced into TMS. An inductive temporal interference stimulation from figure-eight coils achieves deeper and more focused stimulation than that achieved in the aforementioned tradeoff (Xin et al., 2021).

## Neuroscientific Application

Transcranial magnetic stimulation with a figure-eight coil stimulates the human brain within a high spatial resolution. For example, we stimulated the human motor cortex related to the hand and foot areas with a 5 mm resolution. We put electrodes at five hand muscles, such as, abductor pollicis brevis (APB), first dorsal interosseous (FDI), abductor digiti minimi (ADM), brachioradial (BR), biceps brachii (BB), and tibialis anterior (TA) muscles, and we observed motor evoked potentials (MEPs) from these muscles responded to the brain stimulation. We obtained the functional map in this motor cortex (Ueno et al., 1990). The distance between grid points in the functional map is 5 mm. Each functional area has its optimal direction for brain stimulation. When we stimulate the point related to the thumb with the opposite direction with respect to the optimal direction, the thumb does not respond. In the functional map obtained by the TMS with a figure-eight coil, the regional and directional dependences of excitability reflect gyri and sulci of the brain.

Therefore, TMS with a figure-eight coil enables us to study the dynamic spatiotemporal neuronal network in the human brain non-invasively. We can produce so-called virtual lesion transiently. In other words, TMS with a figure-eight coil can cause a transient disturbance locally in the brain for a short period of time. The virtual lesion approach using TMS is a powerful tool for the studies of dynamic mechanisms of the human brain, since the spatiotemporal information processing in the brain has not yet been well clarified.

Epstein conducted an interesting study on episodic memory using the virtual lesion approach when he visited our laboratory (Epstein et al., 2002). Ten Japanese subjects underwent sequential visual stimuli, which contained 18 sets of simple Kanji words and unfamiliar abstract patterns, and the brain was disturbed by TMS with a figure-eight coil between the visual stimulations. The TMS coil was placed at the left dorsolateral prefrontal cortex (DLPFC), right DLPFC, the central vertex, and off the head as a control. After the set of stimuli, subjects took a test to check the memory correctness of the pair of Kanji and abstract patterns. The results indicate that the right DLPFC has an important role in generating the episodic memory. As in this example, TMS with a figure-eight coil can elucidate the dynamic mechanisms of the human higher brain function.

## Applications to Medicine

Clinical studies have shown that rTMS is effective for various psychiatric and neurological diseases. The use of figure-eight coils has enabled us to efficiently induce electric fields in the target area while minimizing the potential risk of side effects caused by the stimulation of surrounding areas. Magnetic stimulators equipped with figure-eight coils have obtained regulatory approval in many countries and are now commercially available. A guideline for the therapeutic use of rTMS was published, based on a review of clinical studies of rTMS (Lefaucheur et al., 2020). In this guideline, the efficacy of rTMS for various psychiatric and neurological diseases was evaluated, and its efficacy in treating depression, motor stroke, and neuropathic pain were categorized as Level A, indicating definite efficacy.

The United States Food and Drug Administration approved the treatment of depression in 2008. The DLPFC is the primary target in the treatment of depression. rTMS treatment is effective in drug-resistant cases.

The successful treatment of neuropathic pain has also been reported in several clinical studies. Stimulation of the primary motor cortex showed positive effects on treatment. The eccentric figure-eight coil shown in Figure 1F was developed and applied for the treatment of neuropathic pain (Hosomi et al., 2020). Clinical studies have shown that the therapeutic effect strongly depends on the conditions and protocol of stimulation.

## Concluding Remarks

Figure-eight coils were originally invented for achieving non-invasive and focal stimulation of the brain. Additionally, its ability of functional mapping of the brain made a significant contribution to neuroscience. In tandem with neuroscientific applications, the therapeutic application of rTMS has also expanded in recent years. Systematic studies of coils have demonstrated the figure-eight coil’s ability to balance the focality and depth of induced fields. In conclusion, it can be surmised that figure-eight coils will continue to play important roles in both neuroscience and medicine, incorporating further technological updates.

## Author Contributions

SU wrote introduction, focality of figure-eight coils, and neuroscience application. MS wrote structural variation of figure-eight coils, tradeoff between depth and focality, recent approaches to extending depth, and applications to medicine. Both authors contributed to the article and approved the submitted version.

## Conflict of Interest

The authors declare that the research was conducted in the absence of any commercial or financial relationships that could be construed as a potential conflict of interest.

## Publisher’s Note

All claims expressed in this article are solely those of the authors and do not necessarily represent those of their affiliated organizations, or those of the publisher, the editors and the reviewers. Any product that may be evaluated in this article, or claim that may be made by its manufacturer, is not guaranteed or endorsed by the publisher.
